# How primary care addresses vaping in the youth: a systematic literature review

**DOI:** 10.1186/s12875-026-03350-1

**Published:** 2026-05-18

**Authors:** Yasmin Baker, Maneth Warnapala, Devan Wasan, Dhruv Vasooja, Elika Najafi, Kristian Peters, Mario Martínez-Jiménez

**Affiliations:** 1 Imperial Business School, Imperial College London, London, UK; 2 School of Medicine, Faculty of Medicine, Imperial College London, London, UK; 3 School of Medicine, University of Manchester, University of Manchester, Manchester, UK; 4 Barts and The London School of Medicine and Dentistry, Queen Mary University of London, London, UK; 5 Department of Health Policy, Stanford University, Stanford, CA USA; 6 Department of Economics and Public Policy, Imperial Business School, London, UK; 7 Centre for Health Economics and Policy Innovation, Centre for Paediatrics and Child Health, Imperial College London, London, UK

## Abstract

**Background:**

Vaping among youth has emerged as a significant public health concern. Primary care professionals are uniquely positioned to address this issue, but their practices, perceptions, and preparedness remain poorly understood.

**Objectives:**

This systematic review aimed to explore how primary care professionals manage e-cigarette use among youth, focusing on three key areas: perceptions, screening practices, and counselling strategies.

**Design:**

This review followed the Preferred Reporting Items for Systematic Reviews and Meta-Analyses (PRISMA) guidelines to ensure rigour and transparency.

**Data sources:**

For studies published from January 2000 to December 2024, a comprehensive search was conducted across Embase, Ovid MEDLINE, Global Health, and Scopus.

**Eligibility criteria:**

Studies focusing on youth (aged 15–24), addressing e-cigarette use within primary care settings, and published in English were included. Exclusion criteria encompassed non-human studies, dual users, and articles without full text.

**Data extraction and synthesis:**

From an initial pool of 896 publications, 14 studies met the inclusion criteria and were analysed. Data extraction followed a structured process, identifying themes related to perceptions, knowledge, screening, and counselling.

**Results:**

Clinicians’ perceptions of e-cigarettes seem to affect their likelihood of discussing usage with patients, while gaps in knowledge and inconsistent screening practices remain common obstacles. Screening rates for e-cigarettes were remarkably lower than those for traditional cigarette products. Counselling practices varied considerably, while strategies such as motivational interviewing and personalised counselling were described, overall practice remained inconsistent. Thus, highlighting missed opportunities for early intervention and the need for more structured and standardised clinical approaches.

**Conclusion:**

Primary care professionals play a crucial role in addressing vaping among youth, but gaps in knowledge, inconsistent practices, and a lack of standardised guidelines limit their effectiveness. Targeted education, clear guidelines, and further research are essential to strengthen primary care interventions in this area.

**Supplementary Information:**

The online version contains supplementary material available at 10.1186/s12875-026-03350-1.

## Introduction

Vaping, defined as the act of inhaling nicotine and other chemicals through electronic cigarettes (e-cigarettes), has rapidly evolved from being a harm-reduction tool for smokers to a widespread behaviour posing significant public health concerns and implications [1]. Although e-cigarettes are often marketed as safer alternatives for adult smokers looking to quit traditional tobacco, their growing popularity among youth (aged 15–24 years old) [2], particularly non-smokers, has raised significant concerns. Recent research indicates that e-cigarettes can impair lung function in young people to a similar degree as traditional smoking, challenging their status as an alternative safer option [3]. Moreover, the long-term health effects of vaping remain uncertain, with concerns regarding respiratory and cardiovascular complications, as well as psychological consequences such as anxiety and depression [4, 5]. This underscores the urgent need for intervention.

In the United Kingdom (UK), the prevalence of e-cigarette usage among young individuals has reached unprecedented levels, with nearly 15% of those aged 16 to 17 having utilised e-cigarettes. Furthermore, almost half of adolescent users indicate a lack of prior exposure to conventional smoking cigarettes [6, 7]. In 2023, 9.1% of the population were estimated to use e-cigarettes, this is compared with 6.2% of the population 5 years earlier in 2018. This shows a percentage increase of 46.8% in 5 years.^6^ Similarly, around one million adults who never smoked regularly now use vape products, with young adults aged 18 to 24 representing a significant portion majority [8]. This significant increase in youth vaping has led to political action, including the UK government’s intention to prohibit the sale of single use vapes which started on 1 June 2025. This was to reduce environmental waste and diminish vaping’s allure for young people [9].

In the NHS, primary care practitioners serve as gatekeepers of the healthcare system, playing a critical role in coordinating patient care and facilitating access to appropriate services [10]. This includes general practitioners (GPs), who function as gatekeepers within the UK system, as well as other professionals such as nurse practitioners and allied healthcare staff. Beyond this, primary care practitioners are central to public health efforts, particularly in the prevention, early identification, and management of substance use behaviours.

Primary care settings also provide a key platform for delivering brief interventions and counselling, including health education and referral to specialist cessation services. These interactions are especially important for younger populations, who may have limited engagement with other healthcare services but frequently access primary care. With this emphasis on prevention, accessibility, and longitudinal care, primary care practitioners are uniquely positioned to address the growing challenge of e-cigarette use among young people.

This systematic literature review aims to synthesise current evidence to explore how primary care professionals perceive, screen for, and counsel youth regarding e-cigarette use, representing the first comprehensive attempt to evaluate how primary care addresses this public health concern in these populations. The findings will provide actionable insights to support clinicians and inform public health policies designed to mitigate the risks associated with vaping in young people. The main research question that we addressed is: How does primary care deal with e-cigarette use in youth populations?

## Methods

The review adhered to the PRISMA (Preferred Reporting Items for Systematic Reviews and Meta-Analyses) guidelines to guarantee methodological rigour and clarity.

### Step 1. identification of relevant studies

#### Data sources

It included a systematic search through four databases: Embase, Ovid MEDLINE, Global Health, and Scopus. To enhance the findings from the systematic review, two individual searches for grey literature were executed using Google Scholar, the King’s Fund, Nuffield Trust, and The British Library.

#### Search strategy

The most recent search was performed in December 2024, ensuring the capture of the latest data literature. No date limits were applied to the search to minimise the risk of inadvertently excluding relevant studies. Descriptors used in the search were in English, derived from keywords and controlled vocabularies, specifically Medical Subject Headings (MeSH) and Health Sciences Descriptors (DeCS), including ‘Electronic cigarette’, ‘ENDS’, ‘Electronic Nicotine Delivery Systems’, ‘Primary care’, ‘General practice’, and ‘Family medicine’. These terms were combined using Boolean operators ‘AND’ and ‘OR’ to enhance the search strategy. Colloquial terminology such as “vape” or “vaping” were not included as standalone search terms. The search strategy prioritised controlled vocabulary to ensure specificity and consistency across databases. These indexing systems are designed to capture relevant articles regardless of informal terminology used in the original text, thereby reducing the risk of missing key studies while limiting the inclusion of irrelevant results. Additionally, restricting the search to standardised terminology improved the precision of results and reduced noise associated with non-specific colloquial language.

The specific search terms, along with the inclusion and exclusion criteria, are detailed in Tables A and B of Supplementary Material 1, respectively.

For grey literature searches, natural language terms (uncontrolled vocabularies) were utilised to ensure a comprehensive and sensitive retrieval of relevant materials.

### Step 2: selection of studies

**Fig. 1:**
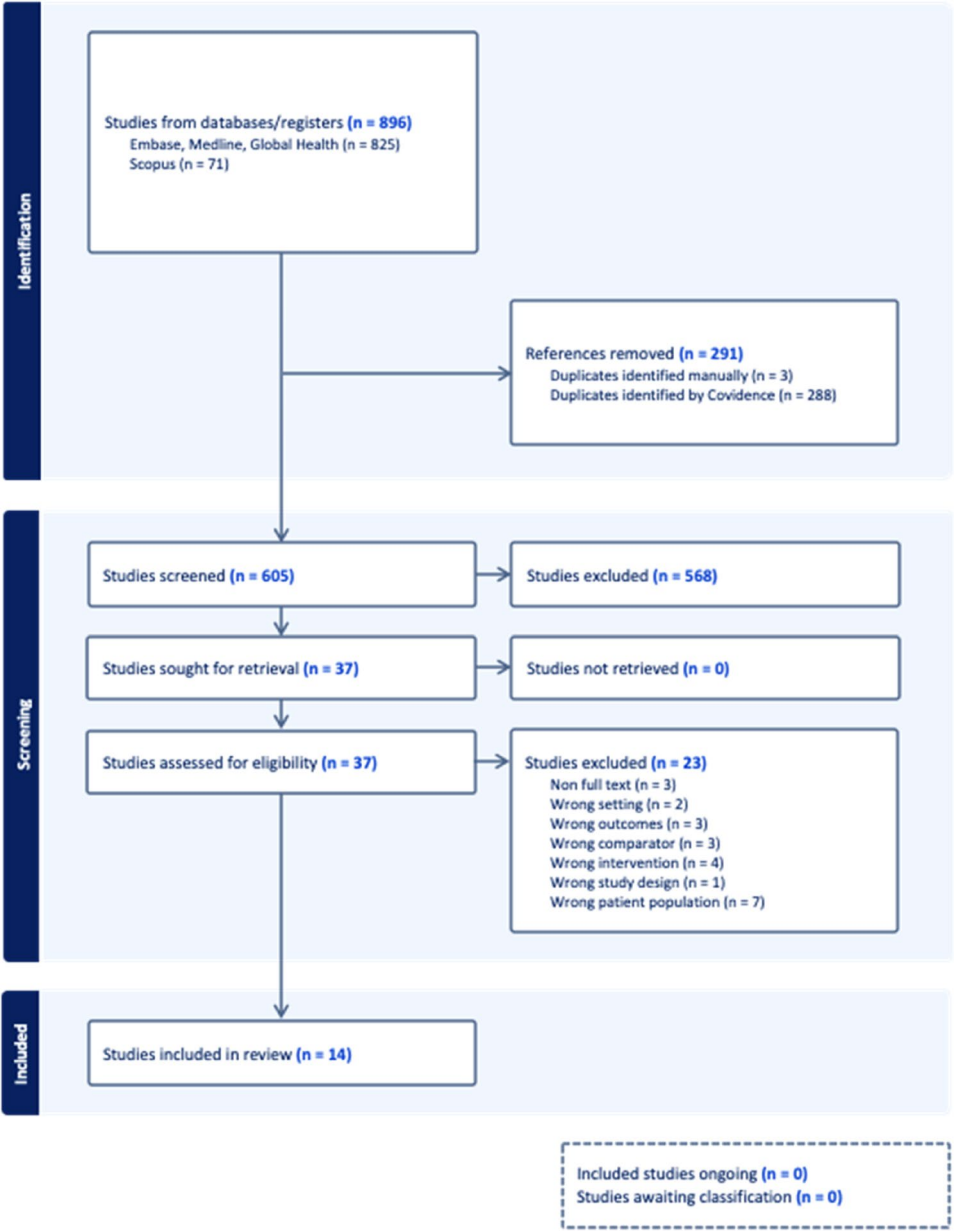
PRISMA Flow Diagram

The search results were imported into Covidence for systematic management, and duplicate records were removed. Two reviewers independently screened the titles and abstracts against the predefined inclusion criteria, resolving any conflicts through discussion. The full-text screening was subsequently conducted independently by the same reviewers to finalise the selection of studies. Reviewers documented reasons for exclusion at the full-text stage, as depicted in the PRISMA flowchart (Fig. 1).

### Eligibility criteria (inclusion)

Studies were included if they involved: (a) young people, including adolescents and young adults (12–25); (b) participants who had never smoked traditional cigarettes; (c) current users of e-cigarettes; and (d) studies conducted in primary healthcare environments.

### Exclusion criteria

Studies were excluded based on the following: (a) involvement of participants who previously smoked or concurrently used conventional tobacco; (b) studies examining e-cigarettes explicitly for smoking cessation purposes; (c) articles without full-text availability; (d) research published in languages other than English; and (e) studies conducted on animals. Only English-language studies were included to ensure conceptual comparability with the UK context, as English-language literature largely reflects healthcare systems with similar primary care structures (e.g. UK, USA, Canada, Australia, New Zealand). This restriction was applied to preserve the relevance and interpretability of findings across comparable clinical and organisational settings, rather than for reasons of feasibility. However, to ensure comprehensive representation of medical professionals’ perspectives on e-cigarette use, research that included adult populations was not excluded.

### Search results

The initial search produced 896 papers, with 605 remaining after duplicates were removed. During the title and abstract screening, we excluded 568 studies that did not meet the defined criteria. An additional 23 studies were removed following full-text screening for being irrelevant or not fulfilling the inclusion requirements. We included 14 full texts for thorough analysis. The complete PRISMA flowchart that details this process can be found in Fig. 1.

The grey literature search did not uncover any pertinent unpublished data. This limitation can be attributed in part to the majority of research on e-cigarettes being included within larger studies focused on conventional cigarette cessation, which fell outside our exclusion criteria. Nevertheless, the thorough search process provided a comprehensive and accurate representation of the existing evidence regarding e-cigarette use in primary care.

### Risk of bias assessment

No studies were excluded on the basis of quality; rather, the bias assessment guided the interpretation and contextualisation of the findings. The ROBINS-I tool assessed intervention studies, while the ROBINS-E tool focused on exposure studies, examining criteria such as participant selection, confounding variable control, and outcome measurement. Studies were categorised as having low, moderate, or high bias risk. All five intervention studies were classified as moderate risk, mainly to do with bias affecting confounding factors. All four exposure studies were considered moderate risk, mainly due to uncontrolled basic socio-demographic confounding factors.

Six included studies were not eligible for a risk of bias assessment using ROBINS-I or ROBINS-E due to their design and purpose. Given the heterogeneity of study types among the lack of causal inference aims, the above tools were not applied to those studies in this review. A detailed summary of risk of bias assessment is provided in Supplementary Material 1 Table C.

### Certainty of the body of evidence

Following the assessment of individual study quality, the overall certainty of the body of evidence for each outcome was evaluated using the GRADE framework to determine how confidently conclusions could be drawn from the included studies. All included studies were observational and therefore began at a starting level of Low certainty. Each outcome was evaluated across the five GRADE domains: risk of bias, inconsistency, indirectness, imprecision and publication bias. Certainty was rated down where serious or very serious concerns were identified and rated up where large, consistent effects strengthened confidence. Final certainty ratings were categorised as High, Moderate, Low or Very low.

One outcome (screening practices and documentation practices) was upgraded. This decision was based on the presence of a large and consistent effect observed across multiple large datasets, including objective electronic health record data, which strengthened confidence in the reliability of this finding. A detailed summary of the certainty of the body of Evidence is provided in Supplementary Material 1 Table D.

### Step 3: data mapping and extraction

Data extraction was conducted using a structured data extraction form developed electronically in Covidence, based on a predefined conceptual framework that included study characteristics, key findings, and thematic patterns related to e-cigarette use in primary care. This framework was informed by preliminary scoping of the literature, with initial themes identified during this stage and iteratively refined following familiarisation with the included studies to ensure relevance and completeness. Two researchers independently performed data extraction to ensure accuracy and consistency, resolving discrepancies through discussion. Extracted data were then exported to Microsoft Excel for management and descriptive analysis, as detailed in Supplementary Material 1 Table E.

### Step 4: compilation, summary and reporting of results

Data were extracted and summarised according to four predefined outcomes: perceptions, knowledge, screening practices, and counselling strategies. Perceptions were defined as primary care professionals’ attitudes and beliefs regarding e-cigarettes, while knowledge referred to their understanding of the health implications, device types, and chemical constituents associated with vaping. Screening practices encompassed methods and frequency of assessing e-cigarette use among youth in primary care settings, and counselling strategies involved the methods clinicians used for communicating and intervening to address vaping behaviours.

A convergent narrative synthesis approach was conducted, integrating qualitative and descriptive quantitative findings. Quantitative results were descriptively summarised and interpreted alongside qualitative data, allowing for the identification of common themes across studies despite heterogeneity in design and outcomes. Studies were grouped by thematic similarity, and intervention characteristics were tabulated to facilitate comparison within each outcome category.

Given the descriptive and qualitative nature of this review, statistical measures such as risk ratios, mean differences, sensitivity analyses, subgroup analyses, or meta-regression were not applicable or conducted. Heterogeneity among study designs and outcomes was qualitatively acknowledged.

## Results

This systematic review analysed 14 studies that met the inclusion criteria, providing insights into how primary care professionals address e-cigarette use among youth. Four major themes emerged from the data synthesis: perceptions, knowledge, screening, and counselling practices.

The studies included in this systematic review comprised a variety of study designs, primarily cross-sectional surveys, qualitative studies, and mixed methods approaches conducted between 2014 and 2024. Studies originated predominantly from the United States, with fewer contributions from the United Kingdom, Australia, and the Netherlands. Populations included primary care clinicians - such as general practitioners, family physicians, and paediatricians - who interacted directly with adolescent and young adult populations regarding e-cigarette usage. Detailed characteristics of each included study, such as author, country, study design, population studied, and key thematic focus, are provided in Supplementary Material 1 Table E.

### Perceptions

Perception, defined as the organisation, identification, and interpretation of sensory information for comprehending the surrounding environment [11] plays a critical role in shaping clinical discussions and decision-making. Across the included studies, the perceptions of e-cigarettes among primary care professionals were consistently found to be complex and varied, shaped by differing interpretations of their risks and benefits relative to conventional tobacco.

A recurring pattern across the evidence base is that clinicians tend to evaluate e-cigarettes comparatively against traditional cigarettes rather than assessing their absolute risks. Where clinicians perceived e-cigarettes as safer alternatives to conventional tobacco products, studies found a reduced likelihood of initiating discussions with patients (*r* = − 0.21; *n* = 776; Pepper et al. [12]). Conversely, where clinicians framed e-cigarettes as a potential gateway to tobacco use, they were more inclined to view these conversations as clinically important (*r* = 0.29; Pepper et al. [12]). This comparative framing is further reflected in prescribing attitudes: a follow-up analysis of the same physician sample found that 40% of paediatricians and family physicians would recommend e-cigarettes for smoking cessation, citing perceived relative safety compared to traditional tobacco [13].

However, the body of evidence also demonstrates that e-cigarette discussions in primary care extend beyond simple tobacco comparisons. Peterson et al. highlighted through qualitative inquiry that these conversations present distinct challenges, including unique clinical considerations, communication strategies, and structural barriers not encountered with conventional tobacco counselling [14]. Although some clinicians emphasise comparative harm reduction, others draw attention to the distinct risks associated with e-cigarette use, including uncertain long-term health effects and the ‘gateway effect,’ whereby adolescents who use e-cigarettes may progress to conventional cigarette smoking. Concern about this gateway pathway was identified as a shared motivator across clinicians, irrespective of their broader perceptions of e-cigarette harm [13].

Collectively, these findings indicate that while clinicians share concerns about both the direct health impacts of e-cigarettes and their potential to facilitate tobacco uptake, no consistent perceptual framework exists across the primary care workforce. This variability underscores the need for standardised clinical guidelines that provide a clear evidence base to anchor clinical judgement, rather than leaving professional practice subject to individual interpretation.

### Knowledge

A consistent finding across the included studies is that primary care clinicians report significant knowledge gaps regarding e-cigarettes, and that these gaps directly undermine their confidence in screening and counselling patients. This pattern was evident across different healthcare systems.

Knowledge deficits were most frequently documented in relation to the health effects of e-cigarettes and their constituent components. A cross-sectional study by McGee et al. (*n* = 61) identified that many clinicians lacked knowledge about both the health effects and the different device types of e-cigarettes [15]. Similarly, a cross-sectional survey by Pepper, Gilkey, and Brewer found that 88% of clinicians expressed a desire for more information about e-cigarettes, with particular demand for content on adverse health effects (75%) [12]. A contributing factor identified across several studies is the limited integration of e-cigarette-related content into medical curricula, which leaves clinicians underprepared to address this growing public health issue [15].

Compounding formal knowledge gaps, the evidence suggests that clinicians frequently rely on non-professional sources for e-cigarette information. In the same physician sample, Pepper, McRee, and Gilkey found that 62% of clinicians identified patients as their primary source of e-cigarette information, followed by news stories (39%) and advertisements (37%), with only 24% using professional channels [12]. Despite this, 83% of the same sample admitted knowing ‘little or nothing’ about e-cigarettes. A pilot cross-sectional study of Australian general practitioners by Singh et al. (*n* = 47) similarly found that only 45% could identify all potential constituents of e-cigarettes, and over half (53%) were unaware of available educational resources [16].

The relationship between knowledge and clinical confidence is well-established across these studies. Singh et al. reported that only 9% of GPs felt confident advising adolescents on e-cigarettes, compared with 36% for conventional cigarettes, with 32% expressing no confidence at all [16]. A qualitative program evaluation by Kovach et al. found that providers lacked confidence in referring patients to cessation services due to knowledge deficits [17]. McGee et al. reported that 74% of healthcare providers felt inadequately informed to counsel on e-cigarettes, and 44% were uncertain about available referral options for adolescent patients [15]. Awareness of e-cigarettes does not appear to be sufficient to overcome this discomfort: Pepper, Gilkey, and Brewer found that more than half (53%) of physicians who were aware of e-cigarettes still felt uneasy discussing them with patients [13] while Peterson et al. raised concerns, through their qualitative inquiry, that inadequate knowledge could damage the doctor–patient relationship [14].

Taken together, these findings reveal a consistent pattern of insufficient knowledge, limited confidence, and inadequate access to professional educational resources across primary care settings. This evidence collectively points to an urgent need for targeted, formally integrated comprehensive training programmes to equip clinicians, particularly those working with young people, to engage effectively with e-cigarette-related discussions.

### Screening

Screening for e-cigarette use in primary care was found to be consistently low, variable in method, and complicated by a lack of standardised terminology and documentation systems. The evidence has been organised into four sub-themes: screening rates, questioning methods, terminology and electronic health record (EHR) documentation, and strategies to improve screening.

#### Screening rates

Across studies, screening rates for e-cigarette use fell substantially below those for conventional cigarette use, indicating that e-cigarettes remain under-prioritised in clinical assessments. In a U.S. cross-sectional study of over 134,000 patients, cigarette screening among adults reached 99.5%, compared to only 34.5% for e-cigarettes [18]. Similarly, Pepper, Gilkey, and Brewer found e-cigarette screening rates of just 14%, compared with 79% for conventional cigarettes [13]. In Australia, Singh et al. reported that only 34% of GPs screened children or adolescents for e-cigarette use [16], while a qualitative study by Peterson et al. (*n* = 15) found that nearly half of clinicians reported never discussing e-cigarettes with patients [14]. Ward et al. further emphasised the importance of early intervention, recommending that screening begin with individuals aged 12 and above [19].

#### Screening methods

Several studies examined how the framing and language of screening questions affects identification rates. Liu, Halpern-Felsher, and Harris (n = 514) demonstrated that using colloquial synonyms such as ‘vape’ or ‘e-cig’ can improve screening precision; however, even with these adaptations, their study found a sensitivity rate of only 50% [20]. To address this limitation, Kaliamurthy and Camenga recommended supplementing initial screening questions with follow-up enquiries about frequency of use, device model, and e-cigarette content [21]. Similarly, Young-Wolff et al. (n = ~ 1.2 million EHR records) similarly advocated for inclusive, broad-phrasing questions, for example, asking patients whether they use any e-cigarette or vaping device, to capture a wider range of usage while simultaneously gathering information on nicotine content and device type [22]. Kaliamurthy and Camenga further suggested that the act of asking about vaping may itself function as a brief intervention, potentially prompting cessation by signalling that it is a clinically concerning behaviour [21].

#### Terminology and EHR documentation

Inconsistencies in clinical terminology and EHR documentation were identified as a barrier to accurate identification and tracking of e-cigarette use, though it should be noted that studies examined these issues within their respective national contexts rather than providing a collectively derived assessment. In the United States, Young-Wolff et al. found that the most common terms used in EHRs were ‘e-cig’ (49%), ‘electronic cigarette’ (25%), and ‘vape’ (28%), with variation across systems [22]. In the UK, an exploratory analysis by Tildy et al. identified seven main clinical coding categories for vaping, with ‘electronic cigarette user’ being the most frequently applied [23]. Clinical coding translates medical terminology into internationally recognised codes, providing accurate data that reflects clinical practice patterns and supports informed decision-making [24]. However, the divergence in terminology both within and across national systems highlights the need for internationally standardised coding frameworks to improve data accuracy and comparability.

#### Strategies to improve screening

Several strategies to enhance screening rates were identified across the evidence. Integrating tailored prompts into EHR systems for clinical histories has been shown to encourage consistent documentation [22]; a cross-sectional quality improvement study of 1,021 young adults by Cano Rodriguez et al. found that incorporating specific e-cigarette screening questions into local EHR systems had the most significant impact on improving identification rates [25]. Additional strategies proposed included expanding the screening responsibilities of healthcare professionals such as registered nurses, offering financial incentives for retrospective clinical coding [23], and establishing standardised EHR prompts to enable longitudinal, population-level monitoring of e-cigarette use and its health effects [22].

### Counselling

Counselling practices for e-cigarette use in primary care were found to be inconsistent and largely reactive. The evidence has been organised into two sub-themes: communication practices and cessation strategies.

#### Communication practices

Survey-based evidence suggests that clinician-initiated discussions about e-cigarettes remain infrequent. Pepper, Gilkey, and Brewer found that physicians initiated e-cigarette discussions 46% of the time, with parents and adolescents accounting for 30% and 23% of initiations respectively, indicating that the onus of raising the topic frequently falls on patients and families [13]. Among UK GPs, self-report data from the pilot cross-sectional study by Singh et al. found that 55% had no structured strategy for discussing e-cigarettes with children or adolescents, and 45% reported finding these discussions difficult [16].

Motivational interviewing was identified as a counselling approach in Peterson et al., in which GPs explicitly described using open-ended questions to explore adolescents’ motivations for vaping, the frequency of their use, and contributing factors such as peer pressure or personal enjoyment [14]. This approach was reported by participants to enable more tailored discussions and to support identification of barriers to quitting. As one example described in the qualitative data, a GP recounted acknowledging a patient’s avoidance of conventional cigarettes while encouraging reflection on the risks of vaping - a strategy combining positive reinforcement with guided self-appraisal. Motivational interviewing is reported here as it was explicitly identified by clinician participants in this qualitative study; whether its use is widespread across primary care settings was not examined in the evidence base. 

#### Cessation strategies

Cessation approaches described across the included studies were varied and often structured around organisational or behavioural frameworks. One model, described by Kovach et al. through their qualitative program evaluation, categorised cessation interventions into three components: creating institutional change by developing a shared vision and securing stakeholder buy-in (drawing on Kotter’s change management framework [26]); establishing consistent clinical processes including systematic screening and quality improvement initiatives; and delivering education to both parents and adolescents on the risks of e-cigarette use [17]. Other studies highlighted the importance of referring adolescent patients to cessation services, offering nicotine replacement therapy, and engaging parents as part of the wider support system around the young person.

Several studies advocated for personalised approaches that support adolescent autonomy in decision-making around cessation [21]. One study additionally proposed discussing the environmental impact of e-cigarette devices as a potential motivator for quitting, particularly given adolescent concerns about sustainability [27].

Collectively, the evidence highlights the need for proactive, structured, and evidence-informed counselling in primary care to address e-cigarette use among young people. However, the variability in reported approaches and the reliance on self-report and qualitative data underscores the limitations of the current evidence base in establishing best practice for cessation counselling in this population.

## Discussion

This systematic review examines how primary care currently manages e-cigarette use among youth, pinpointing essential challenges and improvement opportunities within the interconnected areas of perceptions, knowledge, screening, and counselling. The findings of this review demonstrate that primary care professionals are ideally positioned to tackle e-cigarette use due to their accessibility and frequent interactions with young individuals - a point established in the introduction underpinning this review [10]. Nevertheless, inconsistent practices, along with insufficient knowledge and the absence of standardised guidelines, hinder their capacity to address this escalating public health issue effectively.

Primary care clinicians’ perceptions of e-cigarettes vary significantly, often shaped by comparisons to traditional cigarettes. While some regard e-cigarettes as a safer alternative, which reduces their likelihood of engaging patients in discussions, others are motivated by worries about the gateway effect, prompting more frequent interventions. This concern is supported by external evidence beyond the scope of this review; a longitudinal cohort study in the Netherlands found that increased e-cigarette use among adolescents predicted higher rates of subsequent cigarette smoking [28]. This dichotomy reflects inconsistencies in how clinicians understand and address the risks associated with e-cigarettes, influenced by the lack of a substantial evidence base. Whether these perceptual inconsistencies are directly attributed to a lack of an established evidence base was not explicitly examined in the included studies; this remains an inferential conclusion. A consistent, up-to-date, evidence-informed narrative is essential to ensure that perceptions align with public health priorities. To achieve this, clearer national clinical guidelines, such as those issued by NICE or integrated into Royal College of General Practitioners (RCGP) frameworks, would provide a more authoritative and consistently accessible reference point for primary care professionals than reliance on informal or media-based sources alone.

Clinicians’ lack of knowledge further complicates the situation. Many do not fully understand the health effects of e-cigarettes, the various device types, and the chemical components, often depending on non-expert sources like patients or the media. This dependency points to a shortage of accessible, reliable educational materials. Even with widespread awareness of e-cigarettes, the lack of practical knowledge diminishes clinicians’ confidence in providing counsel and threatens the trust essential to the doctor-patient relationship. To bridge this gap, targeted educational programs are necessary, offering solid, evidence-based information tailored to primary care professionals’ needs.

E-cigarette screening practices are still evolving and vary considerably in application. The rates of screening for e-cigarettes are significantly lower compared to traditional cigarette use. This gap arises from the absence of standardised protocols and varying terminology in EHRs. Beyond these structural barriers, few of the included studies explicitly examined why clinicians do not screen for e-cigarette use. Identified barriers include limited clinician knowledge, unfamiliarity with adolescent vaping behaviours and terminology, and time constraints [14]. However, these were not systematically examined across studies, highlighting an important gap for future research. To improve screening rates, it is essential to incorporate vaping-specific questions into EHR systems, standardise the language used, and refine documentation methods. Additionally, broadening the responsibilities of healthcare professionals, like registered nurses, to include screenings for e-cigarette use may enhance the identification and prompt intervention of related issues.

Effective counselling strategies often seem out of reach, as many clinicians do not have structured methods to discuss vaping, especially with adolescents. Motivational interviewing has surfaced as a valuable technique, allowing clinicians to delve into personal reasons and obstacles related to quitting vaping, such as peer influence or perceived advantages. Customised approaches, like presenting vaping’s environmental effects as a reason to quit, have proven effective in capturing the interest of younger audiences. Nonetheless, the lack of widely adopted frameworks hinders the broader application of these methods. Educational programs that concentrate on cessation techniques and the specific requirements of adolescents could help clinicians feel more equipped to engage in counselling with patients.

**Fig. 2:**
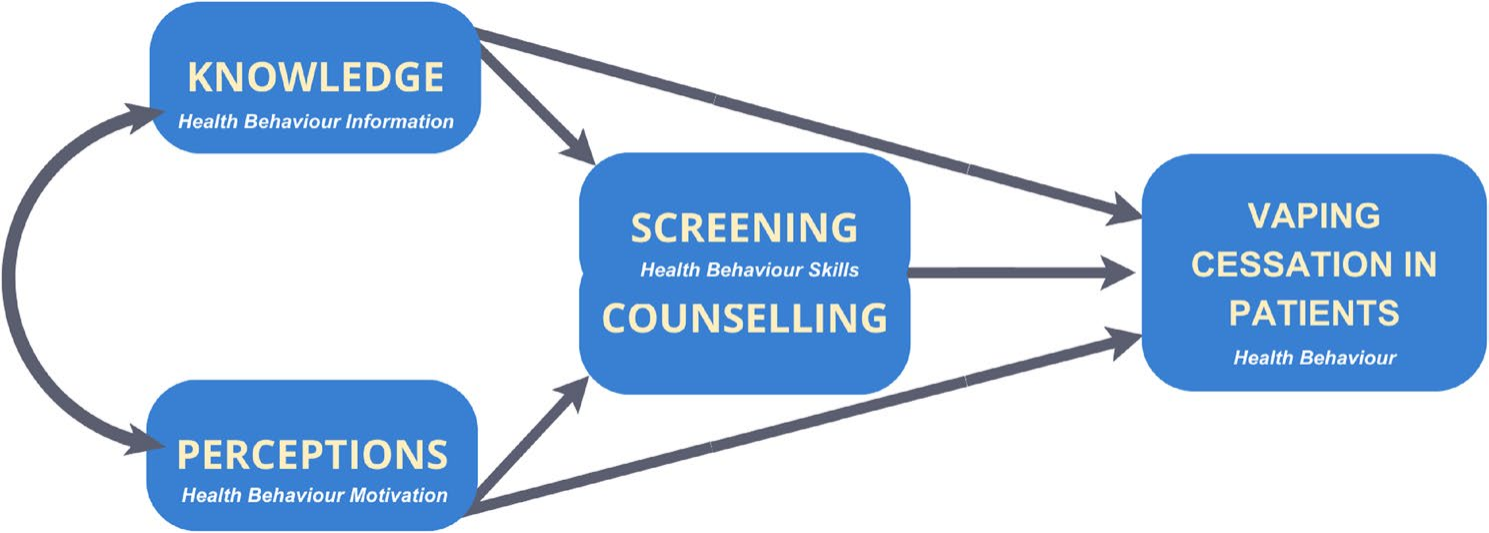
Adapted Information-Motivation-Behavioural Skills (IMB) model applied to primary care management of e-cigarette use

To conceptualise the interplay between these factors, the Information-Motivation-Behavioural Skills (IMB) model provides a valuable framework [29]. This model although originally developed for HIV prevention in patients, has been applied effectively to health behaviour change across a range of settings, including by Zinatsa et al. in the context of frontline healthcare worker behaviour [30]. The IMB model is proposed here as a conceptual lens through which to interpret the current evidence, and as a potential scaffold for future intervention development in primary care, rather than as a framework already applied within the included studies. Each component of the model maps onto the thematic findings of this review: the Information component corresponds to the pervasive knowledge gaps identified across studies, whereby clinicians lacked sufficient understanding of e-cigarette health effects, device types, and cessation options [12, 15, 16]; the Motivation component reflects the variability in clinician perceptions and the inconsistent prioritisation of e-cigarette discussions, shaped by differing views on relative harm and the gateway effect [12, 13]; and the Behavioural Skills component aligns with the identified deficits in structured screening and counselling practice, including the absence of standardised protocols and the limited use of evidence-based communication strategies [14, 16, 17]. Adapted for primary care as illustrated in Fig. 2, the IMB model suggests that equipping clinicians with accurate, accessible knowledge is a prerequisite for building the motivation and practical skills required to address e-cigarette use effectively with young patients.

Despite these insights, several important limitations must be considered. First, the findings’ external validity is constrained by geographic differences. Only two of the 14 studies included in the review were conducted in the UK, while the remaining 12 were from the USA and Australia. The UK-based studies did not explore GPs’ perceptions or practices in depth, with one focusing on EHR codes and the other being a review. As a result, the findings may not represent the practices or perceptions of UK-based primary care professionals or their patient populations, limiting the conclusions’ generalisability. Second, the USA defines primary care professionals as family medicine, general internal medicine, and general paediatrics [31], whereas the UK does not include paediatricians within its primary care framework. This definitional mismatch has substantive implications for interpreting the findings: in the USA, paediatricians may encounter adolescents more frequently and within a developmental health remit that facilitates preventive discussions, whereas UK GPs manage a broader, undifferentiated patient population with shorter consultation times. This difference in scope of practice and frequency of adolescent contact may mean that US-derived findings regarding counselling initiation rates and screening behaviours are not directly transferable to the UK context. Third, the overall certainty of the evidence ranged from very low to moderate. Most outcomes were supported by small, cross-sectional or qualitative studies, resulting in low or very low certainty for perceptions, knowledge and counselling, while evidence for screening was of moderate certainty. These ratings reflect the fragility of the available evidence rather than weaknesses in the review process and underscore the need for more rigorous, UK-specific studies.

Methodologically, this review was limited to English-language publications, which may have introduced language bias. However, this restriction was applied to maintain comparability across health-system contexts rather than convenience; English-language studies predominantly represent systems most analogous to the UK. The inclusion criteria focused on studies involving never-smoker youth within primary care settings, which, while appropriate for the research question, may have limited the generalisability of findings to broader populations. Although a comprehensive search was conducted across Embase, Ovid MEDLINE, Global Health, and Scopus, additional databases such as PubMed, CINAHL, and PsycINFO were not included. While Ovid MEDLINE provides substantial overlap with PubMed, the exclusion of these databases may have limited the identification of the most recent studies and those specific to nursing and behavioural health. This may have reduced the overall comprehensiveness of the review.

Studies including adult populations were included as they provided relevant insights into primary care practitioners’ perspectives and practices. Where possible, data specific to adolescents and young people was extracted. However, only 3 of our 14 studies reported clinician perspectives across mixed-age populations without stratification which may limit the specificity of conclusions specific to those studies.

Finally, although two reviewers independently screened and extracted data, we did not employ formal inter-rater reliability measures, which may have introduced subjective bias in study selection and data extraction. Collectively, these limitations underscore the need for further high-quality, context-specific research - particularly within the UK - to better understand how primary care professionals approach e-cigarette use among young people.

## Conclusion

Primary care’s response to e-cigarette usage in youth remains fragmented, shaped by heterogeneous clinician perceptions, gaps in knowledge, inconsistent screening practices, and largely unstructured counselling approaches. This review identifies key priorities for strengthening primary care practice, including targeted clinician education, the development of more standardised screening processes, and counselling approaches tailored to the needs of adolescents and young adults. Utilising frameworks like the IMB model and emphasising interventions based on evidence can enable primary care to significantly reduce the prevalence of vaping and its risks for youth. Conceptual frameworks such as the IMB model may offer a useful structure through which to organise and develop primary care responses to youth vaping; however, whether such frameworks can meaningfully reduce vaping prevalence in practice remains to be established, and their application in this specific context warrants further empirical evaluation. Future research should focus on UK-specific primary care settings to better understand contextual factors and to inform interventions that are both relevant and transferable across healthcare systems. In particular, there is a need for intervention studies that test the effectiveness of structured educational programmes for clinicians and guideline-development research to establish standardised screening and counselling protocols. Such research would provide the evidence base necessary to move from identifying gaps to implementing and evaluating meaningful change.

## Supplementary Information

Supplementary Material 1.

## Data Availability

All data generated or analysed during this study are included in this published article and its supplementary material files.
