# Supplementary material for: How primary care addresses vaping in the youth: a systematic literature review
Source: BMC Prim Care. 2026 May 18;27:263. doi: 10.1186/s12875-026-03350-1 (PMC13359732; doi:10.1186/s12875-026-03350-1)
Supplement: Supplementary file 1 — Supplementary Material 1. [file 12875_2026_3350_MOESM1_ESM.docx]

**Supplementary Materials**

**Table A.** Search terms for Systematic Literature Review.

| **Search Terms** |
| --- |
| 1. Electronic cigarette/ or vaping/ or e-cig*.mp. |
| 2. ENDS.mp. |
| 3. electronic nicotine delivery systems.mp. |
| 4. E-cigarette.mp. |
| 5. 1 or 2 or 3 or 4 |
| 6. primary care.mp. or primary medical care/ |
| 7. general practice.mp. or general practice/ |
| 8. GP.mp. |
| 9. primary healthcare.mp. or primary health care/ |
| 10. family medicine/ |
| 11. 6 or 7 or 8 or 9 or 10 |
| 12. 5 and 11 |
| 13. limit 12 to English language |
| 14. limit 13 to humans [Limit not valid in Global Health, records were retained] |

**Table B.** Inclusion and Exclusion Criteria for Systematic Literature Review.

| **Inclusion criteria** | **Exclusion criteria** |
| --- | --- |
| Adolescents or young adults* | Previous smokers or dual users |
| Never smokers | E-cigarette use for the purpose of smoking cessation |
| E-cigarette users | Non-full text |
| Primary care | Non-English language |
|  | Non-human studies |

*Notes*: To ensure we captured doctors' perceptions of e-cigarettes in all populations, we opted not to exclude studies focusing on adults.

**Table C.** Risk of Bias Assessment.

**ROBBINS-I**


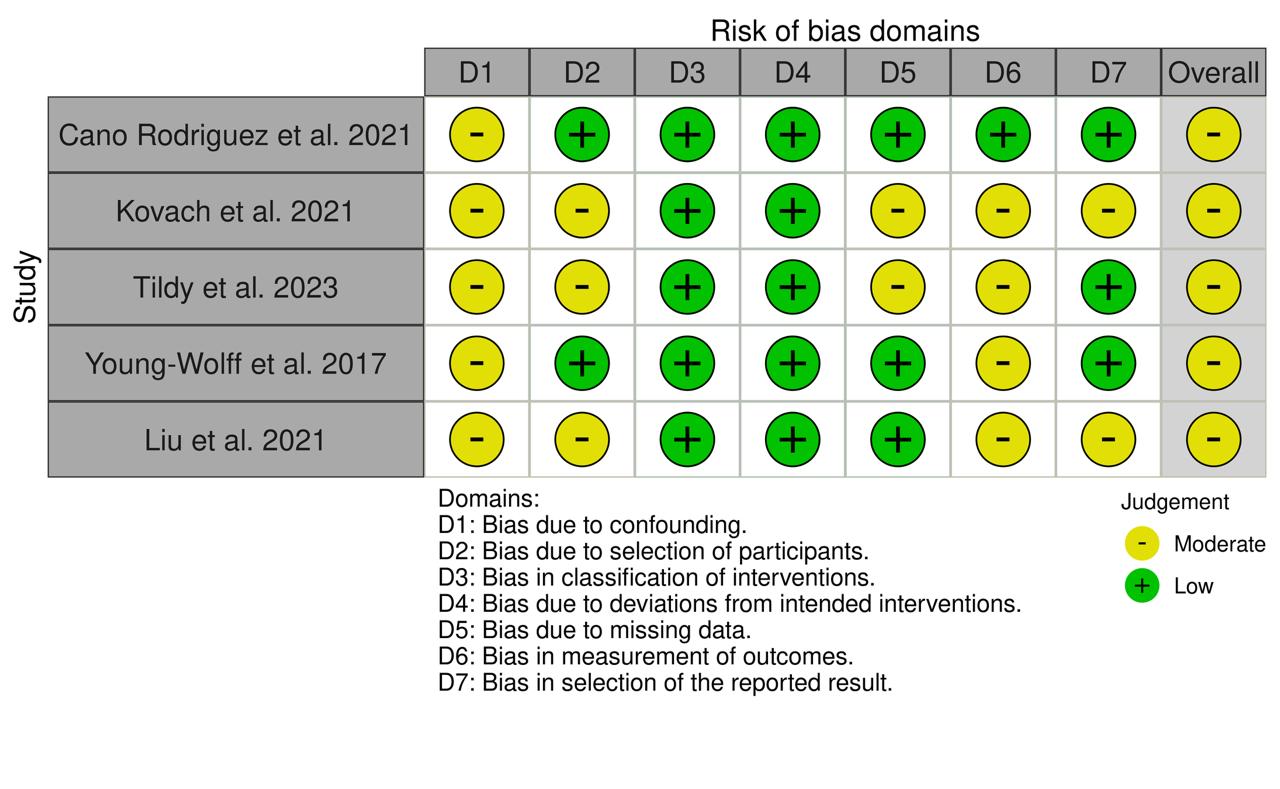

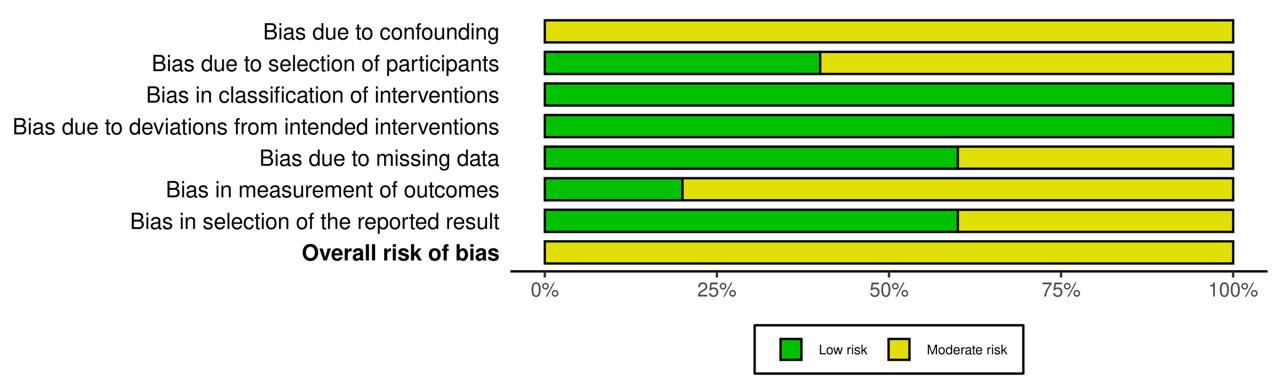


**ROBBINS-E**


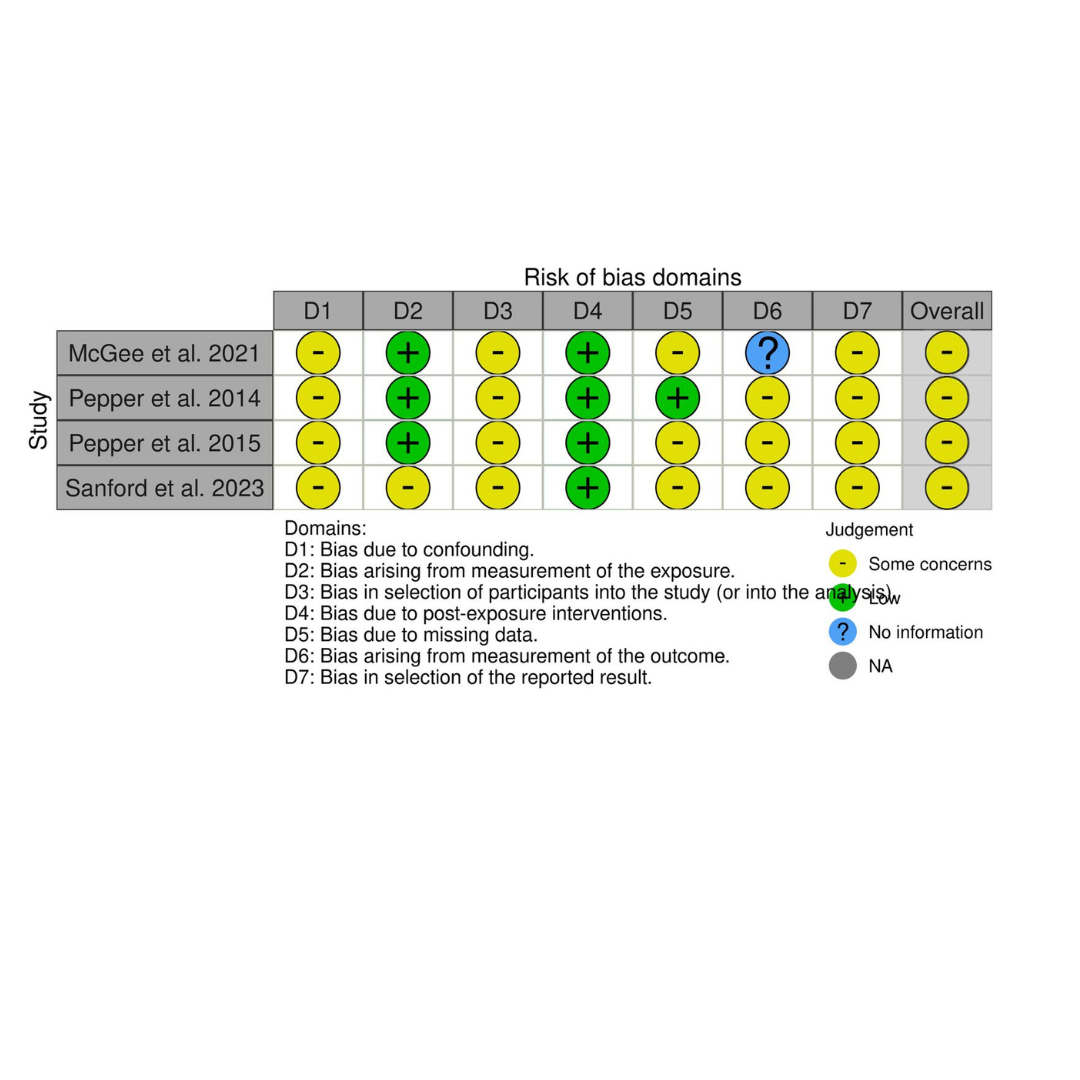


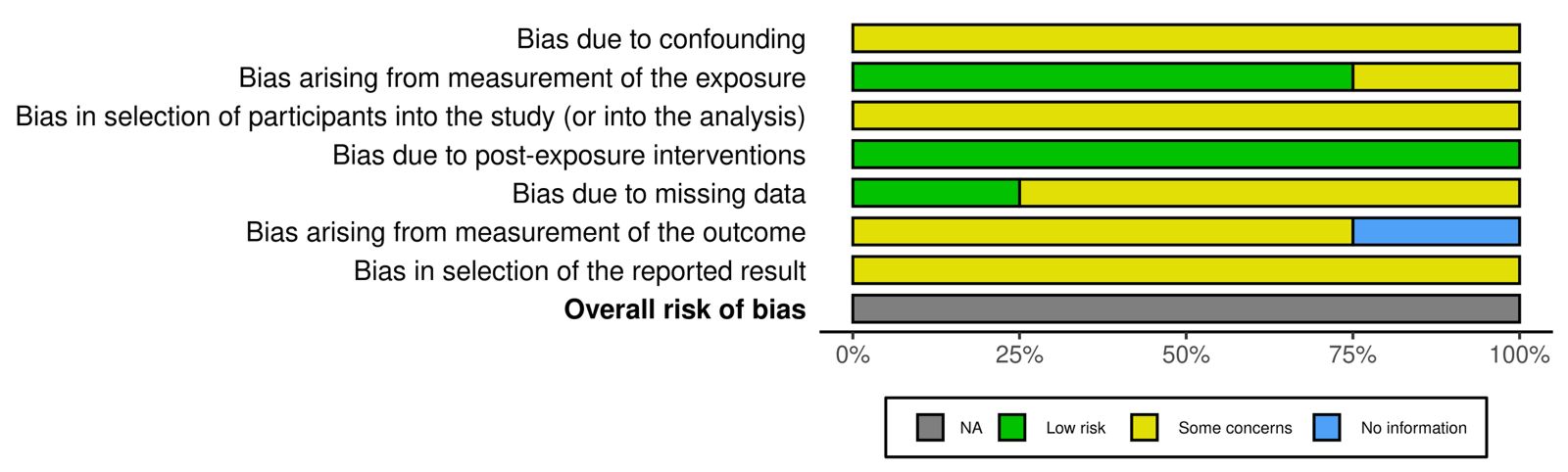


**Table D.** Certainty of the Body of Evidence Table

| Outcome | Studies contributing | Study designs | What the evidence suggests | Starting level | Risk of bias | Inconsistency | Indirectness | Imprecision | Publication bias | Upgrading | Overall certainty | Explanations / footnotes |
| --- | --- | --- | --- | --- | --- | --- | --- | --- | --- | --- | --- | --- |
| Clinicians’ perceptions of e-cigarettes | Pepper 2014; Pepper 2015; McGee 2021; Peterson 2018; Kovach 2021 | Cross-sectional surveys and qualitative evaluation | Views are mixed. Some clinicians see e-cigarettes as harm reduction, others stress harms or gateway risks. Findings vary by setting and specialty. | Low | Serious limitation | Serious inconsistency | Serious indirectness (mostly US paediatric/family medicine; limited UK GP data) | Serious imprecision (small samples; self-report) | Suspected | N/A | **Very low** | Downgraded for bias, inconsistency, indirectness and imprecision. |
| Knowledge of health effects and devices | Kovach 2021; McGee 2021; Singh 2024; White 2023 | Cross-sectional surveys, qualitative, narrative review | Consistent evidence of knowledge gaps about device types, constituents and harms; clinicians express strong need for training. | Low | Serious limitation | Not serious | Serious indirectness (limited UK data) | Serious imprecision (modest samples; few CIs) | Suspected | N/A | **Low** | Downgraded for bias, indirectness and imprecision. |
| Screening and documentation practices | Cano 2021; Liu 2021; Sanford 2023; Tildy 2023; Young-Wolff 2017; Ward 2023; Singh 2024 | EHR analyses, cross-sectional surveys, quality improvement | Screening for vaping is markedly lower than for smoking. The pattern is consistent across large EHR datasets and surveys, including UK CPRD data. | Low | Not serious | Not serious | Minor concern (mix of paediatric and GP settings) | Not serious | Possible | Upgraded one level for large, consistent effect across datasets. | **Moderate** | Upgraded for large, consistent effect and objective measurement; no major downgrades due to dataset size and UK inclusion. |
| Counselling and cessation support | McGee 2021; Ward 2023; Kovach 2021; Peterson 2018; Pepper 2015 | Cross-sectional surveys and qualitative/programme evaluation | Counselling is ad hoc and unstructured. Confidence is low. Referral pathways and guidance are inconsistent. | Low | Serious limitation | Serious inconsistency | Serious indirectness (mostly non-UK; varied primary care definitions) | Serious imprecision (small samples; self-report) | Suspected | n/a | **Very low** | Downgraded for bias, inconsistency, indirectness and imprecision. |

**Table E.** Data Extraction

| # | Title | Authors | DOI | Country | Study Design | Setting/Population | Themes | Subthemes |
| --- | --- | --- | --- | --- | --- | --- | --- | --- |
| 1 | Vaping: Impact of Improving Screening  Questioning in Adolescent Population:  A Quality Improvement Initiative | Cano Rodriguez et al., 2021 | DOI: 10.1097/pq9.0000000000000370 | USA | Quality Improvement | Primary care settings | Knowledge  Screening | Education  Initiation of questions  Tools/Identification methods |
| 2 | Clinical approach to the treatment of e-cigarette use among adolescents | Kaliamurthy & Camenga, 2022 | <https://doi.org/10.1016/>j.cppeds.2022.101203 | USA | Clinical Review | Paediatric care settings | Screening  Counselling | Initiation of questions  Tools/Identification methods  Communication  Cessation |
| 3 | Co-creating opportunities to incorporate  cessation for electronic nicotine delivery  systems in family medicine – a qualitative  program evaluation | Kovach et al., 2021 | <https://doi.org/10.1186/s12875-021-01520-x> | USA | Qualitative Programme Evaluation | Family medicine clinicians | Perceptions  Knowledge  Screening  Counselling | Safety  Education  Confidence  Initiation of questions  Tools/Identification methods  Communication  Cessation |
| 4 | Does Tobacco Screening in Youth Primary Care Identify Youth Vaping? | Liu, Halpern-Felsher & Harris, 2021 | <https://doi.org/10.1016/j.jadohealth.2021.01.017> | USA | Cross-sectional study | Paediatric primary care settings | Screening | Initiation of questions  Tools/Identification methods |
| 5 | Adolescent electronic cigarette counselling: knowledge,  attitudes and perceived barriers among clinical staff in a  primary care setting | McGee et al., 2021 | <https://onlinelibrary.wiley.com/doi/10.1111/jep.13553> | USA | Cross-sectional study | Primary Care physicians | Perceptions  Knowledge  Counselling | Safety  Concern  Education  Confidence  Communication  Cessation |
| 6 | Physicians’ Counseling of Adolescents Regarding E-Cigarette Use | Pepper, Gilkey & Brewer, 2015 | doi:10.1016/j.jadohealth.2015.06.017 | USA | Survey Study | Paediatricians and Family medicine physicians | Perceptions  Knowledge  Screening  Counselling | Safety  Concern  Education  Confidence  Initiation of questions  Communication  Cessation |
| 7 | Healthcare Providers' Beliefs and Attitudes About Electronic  Cigarettes and Preventive Counseling for Adolescent Patients | Pepper, McRee & Gilkey, 2014 | <http://dx.doi.org/10.1016/j.jadohealth.2013.10.001> | USA | Survey Study | Family medicine physicians and Paediatricians | Perceptions  Knowledge  Counselling | Safety  Concern  Education  Confidence  Communication |
| 8 | Pediatric primary healthcare providers’ preferences, experiences and perceived barriers to discussing electronic cigarettes with adolescent patients | Peterson, Fisher & Zhao, 2018 | <https://doi.org/10.1080/17538068.2018.1460960> | USA | Cross-sectional study | Paediatric primary care providers | Perceptions  Knowledge  Counselling | Distinction from cigarettes  Education  Confidence  Communication  cessation |
| 9 | E-Cigarette Screening in Primary Care | Sanford et al., 2023 | <https://doi.org/10.1016/j.amepre.2023.02.030> | USA | Screening study | Primary care settings | Screening | Initiation of questions  Tools/Identification methods |
| 10 | Exploring General Practitioners' Knowledge, Attitudes, and Practices towards E-Cigarette Use/Vaping in Children and Adolescents: A Pilot Cross-Sectional Study in Sydney | Singh et al., 2024 | [10.3390/ijerph21091215](https://doi.org/10.3390/ijerph21091215) | Australia | Cross-sectional study | General practitioners | Knowledge  Screening  Counselling | Confidence  Initiation of questions  Difficulties in communication |
| 11 | How is nicotine vaping product (e-cigarette)  use monitored in primary care electronic  health records in the United Kingdom?  An exploratory analysis of Clinical Practice  Research Datalink (CPRD) | Tildy et al., 2023 | <https://doi.org/10.1186/s12889-023-17200-7> | UK | Data analysis | Primary care records | Screening | Tools/Identification methods |
| 12 | Current Practice to Address Adolescent Vaping in Primary Care | Ward et al., 2023 | <https://doi.org/10.1016/j.nurpra.2023.104751> | USA | Survey study | Primary care clinicians | Screening  Counselling | Initiation of questions  Tools/Identification methods  Communication  Cessation |
| 13 | E-cigarettes in young people:  applying the precautionary principle in primary care | White et al., 2023 | DOI: <https://doi.org/10.3399/bjgp23X734997> | UK | Review | Primary care setting | Knowledge  Screening | Education  Initiation of questions  Tools/Identification methods |
| 14 | Do you vape? Leveraging electronic health records to assess clinician  documentation of electronic nicotine delivery system use among adolescents  and adults | Young-Wolff et al., 2017 | <http://dx.doi.org/10.1016/j.ypmed.2017.08.009> | USA | EHR Analysis | Clinicians using electronic health records from | Screening | Initiation of questions  Tools/Identification methods |

Notes: Study characteristics were extracted using predefined domains including design, setting/population and thematic analysis categories. Subthemes represent inductively or deductively identified elements across studies.
